# Accuracy of pelvic measurements on virtual radiographic projections based on computed tomography scans compared to conventional radiographs pre- and postoperatively

**DOI:** 10.1007/s00402-022-04476-4

**Published:** 2022-06-29

**Authors:** Dominik Kaiser, Armando Hoch, Christoph Stern, Stefan Sommer, Reto Sutter, Patrick O. Zingg

**Affiliations:** 1 Department of Orthopaedics, Balgrist University Hospital, University of Zurich, Forchstrasse 340, 8008 Zurich, Switzerland; 2 Department of Radiology, Balgrist University Hospital, University of Zurich, Zurich, Switzerland; 3 Siemens Healthcare, Zurich, Switzerland; 4 Swiss Center for Musculoskeletal Imaging (SCMI), Balgrist Campus, Zurich, Switzerland; 5 Advanced Clinical Imaging Technology (ACIT), Siemens Healthcare AG, Lausanne, Switzerland

**Keywords:** Developmental dysplasia of the hip, Femoroacetabular impingement, Lateral center edge angle, Acetabular index

## Abstract

**Background:**

The anteroposterior (ap) radiograph of the pelvis is decisive in the diagnosis of different pathologies of the hip joint. Technical advantages have reduced the radiation dose of pelvic CT to levels comparable to radiographs. The purpose of this study was to validate if standard radiographic parameters (lateral center edge angle, medial center edge angle, acetabular index, acetabular arc, extrusion index, crossover sign and posterior wall sign) can accurately be determined on radiograph-like projections reconstructed from the CT dataset pre- and postoperatively.

**Methods:**

A consecutive series of patient with symptomatic dysplasia of the hip and a full radiologic workup (radiographs and CT scan pre- and postoperatively) who underwent periacetabular osteotomy were included. Standard radiographic parameters were compared between radiographs and radiograph-like projections by two authors pre- and postoperatively.

**Results:**

A total of 16 hips (32 radiographs/32 radiograph-like projections) were included in the study. No significant difference was found between the radiographs and radiograph-like images for all parameter for both examiners. ICC between radiograph and radiograph-like projections for all investigated parameters showed good to excellent reliability (0.78–0.99) pre- and postoperatively.

**Conclusion:**

Radiograph-like projections show comparable results to radiographs with regard to the important investigated parameters (lateral center edge angle, medial center edge angle, acetabular index, acetabular arc, extrusion index, crossover sign and posterior wall sign). Thus, ultra-low-dose CT scans may reduce the need for conventional radiographs in pre- and postoperative analyses of 3-dimensional hip pathologies in the future, as the advantages increasingly outweigh the disadvantages.

## Introduction

Radiographs are the standard modality for imaging in orthopedic surgery and are commonly acquired before and after surgical procedures. The anteroposterior (ap) radiograph of the pelvis is decisive in the diagnosis of different pathologies of the hip joint. Various quantitative and semi-quantitative parameters define common pathologies such as developmental dysplasia of the hip (DDH) and increased acetabular coverage in pincer type femoroacetabular impingement (FAI) which are both associated with early development of osteoarthritis [1–4]. Important parameters quantifying acetabular coverage and the orientation of the acetabular roof [1, 5–9] include the lateral and medial center edge angle (LCEA, MCEA), acetabular arc, extrusion index, acetabular index (AI) the crossover sign and the posterior wall sign.

Acetabular coverage and roof orientation can also be quantified by computed tomography (CT) [4, 10, 11]. Literature comparing the measurements on pelvic ap radiographs and CT show intermodality ICC for the CT scans and radiographs of 0.79 [0.61–0.87] indicating a moderate to good reliability [12] and a somewhat lower ICC from 0.43 to 0.8 when comparing MR scans to radiographs [13]. However, the modality of the conventional radiograph (point source of X-ray beams) is substantially different to CT scans (X-ray cone beam distortions which are corrected for during image reconstruction) and thus compares two different entities. Nonetheless, a reliable assessment and deformity analysis as well as an exact corresponding 3D planning of the surgical correction (i.e., periacetabular osteotomy) is highly desired and may be beneficial for the patient.

An unrelated but until recently important disadvantage of CT imaging was the greater radiation exposure in this typically young patient population carrying an increased lifetime risk for malignancy [14].

However, technical updates with tin prefiltration markedly reduced the radiation dose of pelvic CT scans to levels comparable to conventional X-rays without sacrificing image quality with respect to the bone contrast [15].

A logical next step is to exploit technical possibilities in data processing and 3D reconstruction of the obtained CT datasets to generate radiograph-like projections, which could relevantly reduce and/or omit the need for conventional radiographs in the assessment of 3-dimensional hip pathologies in young patients.

While the above-mentioned pelvic parameters can be readily assessed on a radiograph-like projection they have not yet been validated.

Therefore, the purpose of this study was to validate if standard radiographic parameters (LCEA, MCEA, acetabular arc, extrusion index, AI, the crossover sign and the posterior wall sign) can reliably be determined on virtual radiograph-like projections based on CT data of the pelvis and whether they provide comparable results to the parameters obtained from standard ap pelvis radiographs pre- and postoperatively after PAO.

## Materials and methods

This study was approved by our ethical review board (KEK ZH: BASEC Nr. 2018-01921) and all participants gave written informed consent.

### Patient population

A consecutive series of patients with symptomatic developmental dysplasia of the hip and a full radiologic workup (pre- and postoperative radiographs and CT scans) who underwent a periacetabular osteotomy (PAO) from July 2017 to October 2019 were identified. Ethical approval was obtained at the local ethics committee. All the patients gave their informed consent (KEK Zürich, BASEC-Nr. 2018-01921).

### Image acquisition

The pelvic radiograph was obtained supine with internal leg rotation of 15° and a film-focus distance of 120 cm. The center of the X-ray beam was directed to the midpoint of the symphysis and a line connecting the anterosuperior iliac spines [16]. CT were also acquired in the supine position with legs 15° internally rotated and with the following settings: automated tube voltage selection (CARE kV, reference 120 kV) and tube current modulation (CARE Dose4D, reference 147 mAs), a pitch of 0.8, a collimation width of 0.6 mm and a rotation time of 0.5 s. Radiograph-like projections were calculated from the CT data using a customized 3D cone beam projection algorithm based on the implementation from Kim et al. using MATLAB (The MathWorks, Inc., Version R2018b) [17]. CT were also acquired in the supine position with legs 15° internally rotated. The settings of a real pelvic radiograph were simulated with the same center beam and virtual film-focus distance of 120 cm. The generation of virtual radiograph-like projections from the CT data was computed offline with a minimal time expenditure of less than 5 min per image.

### Measurement

The radiographic parameters were independently measured by a board-certified orthopedic surgeon (Examiner 1, D.K.) and a board-certified musculoskeletal radiologist (Examiner 2, C.S.) on the preoperative radiograph and the preoperative radiograph-like projection as well as on the postoperative radiograph and the postoperative radiograph-like projection. The institutional picture archiving and measurement system (Phönix PACS GmbH, Freiburg im Breisgau, Germany) was used for all measurements.

The angles were measured as defined in Table 1 [9].

**Table 1:** Definitions of the investigated radiographic hip parameters

Lateral center edge angle (LCEA)	Angle formed by a line parallel to the longitudinal pelvic axis and a line connecting the center of the femoral head with the lateral edge of the acetabular sourcil
Medial center edge angle (MCEA)	Angle formed by a line parallel to the longitudinal pelvic axis and a line connecting the center of the femoral head with the medial edge of the acetabular sourcil
Acetabular arc	Angle formed by two lines connecting the center of the femoral head with the medial and the lateral edge of the acetabular sourcil (sum of the LCE angle and the MCE angle)
Extrusion index	Percentage of uncovered femoral head in comparison to the total horizontal head diameter
Acetabular index	Angle formed by a horizontal line and a line through the most medial point of the sclerotic zone of the acetabular roof and the lateral edge of the acetabulum
Crossover sign	Positive if the projected anterior wall crosses the posterior wall
Posterior wall sign	Positive if the posterior acetabular rim is projected medial of the center of the center of the hip

## Statistical analysis

Statistical analysis to determine the difference between the measured parameters on the radiograph and the radiograph-like projection for each examiner was performed using the Wilcoxon test. Differences were considered to be statistically significant for *p* values < 0.05. The ICC (Intraclass Correlation Coefficient) for the different parameters were calculated using SPSS 27.0 (IBM, Armonk, NY, USA) between the two examiners (Orthopedic surgeon and MSK radiologist) as well as between the two image entities (radiograph and radiograph-like projection). Values of < 0.5 indicate poor reliability, 0.5–0.75 moderate reliability, 0.75–0.9 indicate good reliability and values > 0.9 indicate excellent reliability.

## Results

**Fig. 1:**
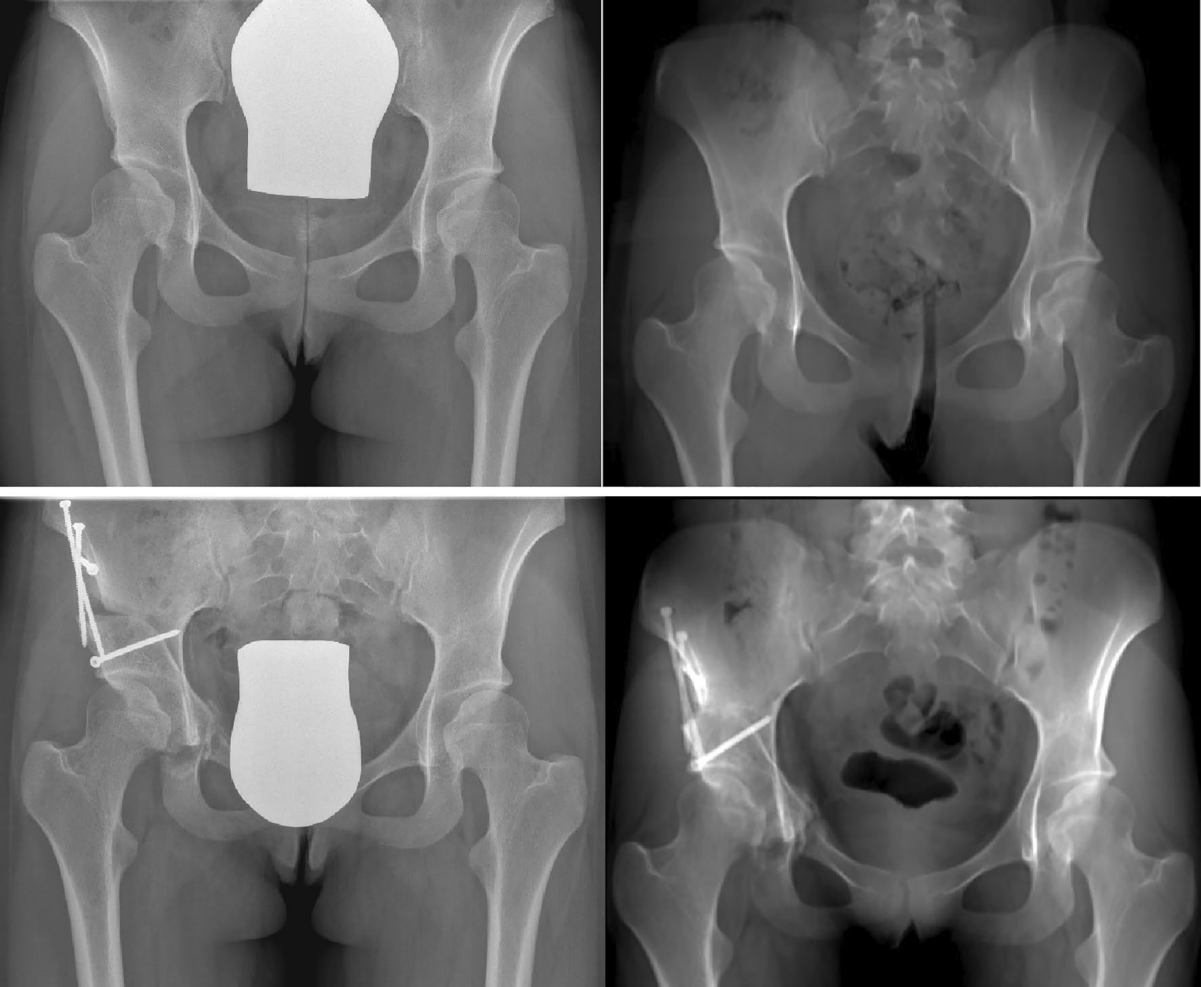
Side-by-side comparison of conventional radiographs on the left and radiograph-like reconstructions on the right depicting the excellent quality

A total of 16 hips (15 patients) were included with an average age of 26 years (range 15–35 years) at the time of surgery. A total of 32 radiographs (16 preoperative/16 postoperative after PAO) and 32 radiograph-like projections based on CT examinations (16 preoperative/16 postoperative after PAO) were analyzed (Fig. 1). The mean differences of the measured parameters are summarized in Table 2. There was no significant difference between the measured parameters (LCEA, MCEA; acetabular arc, Extrusion index, ACI) between the measurements obtained from the radiograph and the radiograph-like reconstruction for both examiners pre- and postoperatively (Table 3). The ICC’s are summarized in Table 4. All measured parameters showed a showed a good (0.75–0.9) to excellent reliability (> 0.9) when comparing the LCEA, MCEA, acetabular arc, extrusion index and ACI between the two image modalities for both examiner 1 and examiner 2 pre- and postoperatively. When comparing the values between the two examiners a good to excellent reliability was seen for LCEA, MCEA, acetabular arc and ACI pre- and postoperatively, while a moderate reliability was seen between the extrusion index preoperatively on radiographs and radiograph-like images (0.65/0.53) and a poor reliability was seen for the extrusion index postoperatively on radiographs and radiograph-like images (0.32/0.38).

**Table 2:** Overview of measured values

	Examiner I				Examiner II			
	Preoperative		Postoperative		Preoperative		Postoperative	
	Mean	SD	Mean	SD	Mean	SD	Mean	SD
Mean values radiographs								
LCEA [°]	17.52	1.89	33.75	3.544	17.64	1.95	33.49	4.19
MCEA [°]	41.93	5.33	28.92	5.31	42.19	5.45	28.79	6.22
Acetabular arc [°]	59.45	4.61	62.67	5.13	59.84	5.03	62.28	6.71
Extrusion index	29.45	4.30	13.44	3.64	28.14	5.39	14.31	4.55
AI [°]	14.75	3.88	0.47	3.36	14.95	3.25	1.3	4.00
Cross over sign^a^	6		4		6		4	
Posterior wall sign^a^	14		11		15		8	
Mean values radiograph-like projections								
LCEA [°]	17.60	2.36	33.49	3.52	17.91	2.11	33.34	4.12
MCEA [°]	42.07	5.48	28.54	4.86	42.26	6.01	27.97	5.89
Acetabular arc [°]	59.68	4.69	62.03	4.06	60.17	5.81	61.31	6.01
Extrusion index	28.89	3.70	13.59	3.99	28.37	4.83	14.29	4.24
AI [°]	14.18	4.28	0.29	3.56	14.72	3.53	1.28	3.84
Cross over sign^a^	6		4		6		4	
Posterior wall sign^a^	14		11		15		9	

^a^Number of patients with positive cross over sign/posterior wall sign

**Table 3:** Overview of statistical analysis

*n* = 16	LCEA	MCEA	Acetabular arc	Extrusion index	AI
(a) Comparison of radiograph and radiograph-like projection preoperative (Examiner 1)					
*P* = *	0.73	0.54	0.47	0.44	0.059
(b): Comparison of radiograph and radiograph-like projection preoperative (Examiner 2)					
*P* = *	0.22	0.87	0.96	0.93	0.254
(c): Comparison of radiograph and radiograph-like projection postoperative (Examiner 1)					
*P* = *	0.30	0.54	0.50	0.76	0.63
(d): Comparison of radiograph and radiograph-like projection postoperative (Examiner 2)					
*P* = *	0.84	0.36	0.35	0.83	n.a.^a^

^*^Wilcoxon test

^a^*N* = 8, as 8 values = 0, no analysis possible

**Table 4:** Summary of ICC

*n* = 16	LCEA	MCEA	Acetabular arc	Extrusion index	AI	Crossover sign^b^	Posterior wall sign^b^
(a) ICC^a^ between radiographs and radiograph-like projections (Examiner 1)							
Preoperative	0.91	0.97	0.94	0.88	0.97	0 (0%)	0 (0%)
Postoperative	0.88	0.923	0.85	0.94	0.96	0 (0%)	0 (0%)
(b) ICC^a^ between radiographs and radiograph-like projections (Examiner 2)							
Preoperative	0.87	0.96	0.94	0.78	0.97	2 (13%)	0 (0%)
Postoperative	0.96	0.87	0.84	0.84	0.99	0 (0%)	1 (6%)
(c) ICC^a^ between Examiner I and II radiographs							
Preoperative	0.85	0.96	0.89	0.65	0.91	0 (0%)	1 (6%)
Postoperative	0.92	0.94	0.88	0.38	0.87	2 (13%)	3 (19%)
(d) ICC^a^ between Examiner I and II radiograph-like projections							
Preoperative	0.83	0.91	0.82	0.53	0.91	2 (13%)	1 (6%)
Postoperative	0.89	0.85	0.76	0.32	0.85	2 (13%)	2 (13%)

^a^ICC: < 0.5: poor reliability, 0.5–0.75: moderate reliability, 0.75–0.9: good reliability, > 0.9: excellent reliability

^b^Number of different interpretations

## Discussion

The aim of this present study was to determine if standard radiographic parameters (LCEA, MCEA, acetabular arc, extrusion index, AI, the crossover sign and the posterior wall sign) can reliably be determined on virtual radiograph-like projections based on CT data and if they provide comparable results to the parameters obtained from standard ap pelvis radiographs and if these measurements cannot only be reliably determined on CT scans of native pelvises but also postoperatively after PAO.

With this study, we could confirm our hypotheses. There was no significant difference between the measurements for LCEA, MCEA, acetabular arc, Extrusion index, AI between the radiographs and the radiograph-like images pre- and postoperatively for both examiners (Table 3).

The intraclass correlation coefficient showed good to excellent reliability (ICC 0.84–0.99) when comparing the results of the measured parameters between radiograph and radiograph-like projections for both examiners pre- and postoperatively. A good to excellent ICC was seen between the two examiners as well for all parameters (ICC 0.76–0.96) pre- and postoperatively, except the extrusion index (poor to moderate reliability). These results are comparable to the literature where radiographic studies have shown that the extrusion index has a greater variability and less reliability than the other measurements [12, 18, 19].

Assessment of the crossover sign and posterior wall sign showed minimal variation between radiographs and radiograph-like projections (0–13%) and a slightly greater variation between the two examiners (0–19%). The overall comparability is very good. The differences were seen in two patients with a small cranial cross over sign.

In view of these results, in our opinion, it is justifiable to perform only a CT scan for preoperative planning of a periacetabular osteotomy and postoperative follow-up. Admittedly, in everyday clinical life, a conventional radiograph has often been obtained in most patients prior to our consultation. To change this standard will of course take some time and require increased awareness of the technical possibilities and dose reduction potential of tin prefiltration in ultra-low-dose CT. In an in-house study, we have shown that tin-filtered ultra-low-dose pelvic CT produce excellent images depicting anatomy and osseous pathologies comparable to standard CT, while substantially reducing the median effective dose to 0.38 mSv per patient [15]. Albeit, this study was performed with standard CT scans, we are convinced that the results would be equal with ultra-low dose CT image data.

The driving force to change the standard of procedure is the overall dose reduction that can be achieved in this typically young patient collective while maintaining the quality and accuracy of the assessment of the mentioned parameters combined with greater precision in the assessment of osseous consolidation. Thus, the number of necessary examinations can be reduced by omitting our routine conventional radiographs (pelvis ap and cross-table axial view). This does not only reduce the radiation dose but also the administrative effort, the time required for the patient and the infrastructure, as well as the costs. Further downsides of the conventional radiographs are the projection-based and examiner-dependent technique. The radiograph is prone to rotational as well as centering errors potentially leading to a repetition of the examination.

The use of CT as a standard procedure allows three-dimensional deformity analysis, 3D planning, but also 3D quantification of the achieved surgical result. With the CT dataset, not only a pelvic ap can be reconstructed. Additional reconstruction options include cross-table radiographs and false-profile images [20] which can be used for further measurements such as the anterior center edge angle [21]. As mentioned before, a further benefit includes the precise analysis of the osseous consolidation of the osteotomies. In summary we are convinced that the implementation of ultra-low dose CT examinations with calculation of radiographic projections may positively influence the overall quality of care while reducing radiation dose for our patients. The biggest disadvantage of the technique currently remains a time expenditure of less than 5 min per examination. Within this study, the radiograph-like projections were generated by a board-certified radiologist (C.S.) but can readily be instructed to be performed by a radiologist assistant. Nevertheless, the additional time expenditure is readily justified if (a) an additional radiograph can be omitted reducing administrative effort and cost and (b) the radiation exposure of our patients can be reduced. In addition, the development of automated algorithms for producing the radiographic projections will allow to accelerate the workflow substantially in the future.

In summary, we believe that by reconstructing radiograph-like projections from tin-filtered ultra-low dose CT scans, we can maintain the diagnostic quality in cases of DDH and FAI due to acetabular overcoverage and acetabular retroversion while substantially reducing radiation exposure to a median effective dose of 0.38 mSv, which is not only 84% lower compared to standard CT scans as noted by Stern et al.[15] but also comparable to a pelvic ap and cross-table radiograph at our institution with a mean effective dose of 0.34 mSv [unpublished results].

There are limitations to this study. The number of participants was low, although we do not believe a greater number will change the clear findings and the achieved accuracy which was good to excellent. We have focused on parameters obtained from the pelvic ap radiograph as they are most clinically relevant.

## Conclusion

Radiograph-like projections show comparable results to radiographs with regard to the important investigated parameters. Thus, ultra-low dose CT scans may reduce the need for conventional radiographs in pre- and postoperative analyses of 3-dimensional hip pathologies in the future as the advantages increasingly outweigh the disadvantages.
